# Resting-state functional connectivity and cortical thickness characterization of a patient with Charles Bonnet syndrome

**DOI:** 10.1371/journal.pone.0219656

**Published:** 2019-07-18

**Authors:** Charlotte Martial, Stephen Karl Larroque, Carlo Cavaliere, Sarah Wannez, Jitka Annen, Ron Kupers, Steven Laureys, Carol Di Perri

**Affiliations:** 1 GIGA-Consciousness, University of Liège, Liège, Belgium; 2 Coma Science Group, University Hospital of Liège, Liège, Belgium; 3 NAPLab, IRCCS SDN Istituto di Ricerca Diagnostica e Nucleare, Naples, Italy; 4 BRAINlab, Department of Neuroscience, Panum Institute, University of Copenhagen, Copenhagen, Denmark; 5 Unité COSY, Institute of Neuroscience, Université Catholique de Louvain, Brussels, Belgium; 6 Harland Sanders Chair in Visual Science, School of Optometry, University of Montreal, Montreal, Quebec, Canada; 7 Centre for Clinical brain Sciences, Centre for Dementia Prevention, IK Dementia Research Institute, University of Edinburght, Edinburgh, United Kingdom; Nathan S Kline Institute, UNITED STATES

## Abstract

Charles Bonnet syndrome (CBS) is a rare condition characterized by visual impairment associated with complex visual hallucinations in elderly people. Although studies suggested that visual hallucinations may be caused by brain damage in the visual system in CBS patients, alterations in specific brain regions in the occipital cortex have not been studied. Functional connectivity during resting-state functional magnetic resonance imaging (rs-fMRI; without hallucinations) in CBS patients, has never been explored. We aimed to investigate brain structural and functional changes in a patient with CBS, as compared with late blind (LB) and normally sighted subjects. We employed voxel-based morphometry and cortical thickness analyses to investigate alterations in grey matter characteristics, and rs-fMRI to study changes in functional brain connectivity. Decreased grey matter volume was observed in the middle occipital gyrus and in the cuneus in the CBS patient, and in the middle occipital gyrus and in the lingual gyrus within LB subjects, compared to their respective control groups. Reductions in cortical thickness in associative and multimodal cortices were observed in the CBS patient when comparing with LB subjects. The precuneus exhibited increased functional connectivity with the secondary visual cortex in the CBS patient compared to the controls. In contrast, LB patients showed decreased functional connectivity compared to sighted controls between the DMN and the temporo-occipital fusiform gyrus, a region known to support hallucinations. Our findings suggest a reorganization of the functional connectivity between regions involved in self-awareness and in visual and salience processing in CBS that may contribute to the appearance of visual hallucinations.

## Introduction

Charles Bonnet syndrome (CBS) is a rare syndrome characterized by complex and recurrent visual hallucinations in people without psychological disorder. This syndrome typically occurs in people who have a visual deficit, generally resulting from macular degeneration, optic neuritis, cataracts, retinitis pigmentosa, glaucoma or diabetic retinopathy [1]. Typically, the experienced visual hallucinations are clearly recognized as unreal by CBS patients. So far, CBS has been associated with lesions of the visual system ranging from the retina to the occipital cortex [2]. Previous magnetic resonance imaging (MRI) studies have provided diverging results about the presence of structural brain alterations in CBS patients. Some studies have suggested that visual hallucinations in CBS patients may be caused by structural damage somewhere within the visual system [3–7], even though the specific regions have not been determined. Functional neuroimaging underlying the pathophysiology of visual hallucinations in CBS subjects remains relatively unexplored. Functional studies in actively hallucinating CBS patients have reported increased activation of the ventral occipital lobe and the inferior lateral temporal cortex (mainly the fusiform gyrus) during spontaneous hallucinations [3, 8–10]. However, functional connectivity during resting state functional magnetic resonance imaging (rs-fMRI), i.e. without active hallucinations in CBS patients, has never been reported. Resting state may offer an ideal approach for providing novel insights into the functional pathways that allow the emergence of visual hallucinations.

Here, we investigated a late-onset visually-impaired patient with CBS who experiences complex visual hallucinations with a structural-functional imaging approach. In order to discern the findings strictly related to the visual impairment from those related to visual hallucination in our patient, we included three control samples for comparison: one consisting of a group of normal sighted subjects with the CBS patient, a second one consisting of a group of late blind (LB) subjects without CBS, and a third group of normal sighted subjects matched in age and sex with the LB. We used voxel-based morphometry (VBM) to study differences in grey matter density, and cortical thickness analysis to investigate structural cortical rearrangement in CBS (given that the present patient suffers from a degenerative eye disease, we focused on the lateral geniculate nucleus (LGN) from the visual pathways). We further investigated functional connectivity patterns by means of rs-fMRI in order to detect changes in functional connectivity patterns underlying this syndrome. To this end, we used both a seed-based approach focusing on brain areas implicated in visual consciousness and salience networks.

## Materials and methods

### Participants

#### Case report

An 85-year-old man with visual impairment and no psychiatric history presented to the neurological department of the University Hospital Centre of Liège (Belgium). He reported his five-year history of increasing frequency of visual hallucinations and was able to give a detailed and coherent description of his hallucinatory experiences. The patient suffered from retinitis pigmentosa, a degenerative eye disease causing severe vision impairment due to progressive degeneration of the rod photoreceptors in the retina [11]. At the age of 3 years, he began experiencing progressive and gradual loss of vision. During his adolescence, his peripheral field of vision progressively narrowed (progressive development of "tunnel vision") and he developed hemeralopia, i.e. night vision deterioration by the abolition of rod cells. At the age of 70 he also lost central vision which resulted in complete blindness at the age of 80. The patient reported a positive family history of CBS.

This man started experiencing visual hallucinations at the age of 80. Visual hallucinations gradually became more frequent and occurred many times during the day. At the time of the visit, he repeatedly described seeing bilateral visual hallucinations with vivid details. The hallucinations reported by this patient were well formed, ranging from simple flashes or colored background to more complex with the appearance of common faces, objects and bodies of people, or landscapes. The hallucinations varied in size and color, and were binocular, covering the entire visual field. However, animations (i.e. scenes in motion) were only present in the right visual field. The visual hallucinations were always perceived as pleasant; they generally occurred with the eyes open and did not disappear when closing the eyes and were never accompanied by abnormal perception in any other sensory modality. The patient was fully aware of their unreal nature but he was not able to consciously control their occurrence or content. Based on his clinical history and the diagnostic exams he underwent, a diagnosis of CBS was made by the neurologist. Indeed, the patient fulfilled the four diagnostic criteria for CBS: (1) hallucinations must be complex, repetitive, and persistent; (2) awareness that the hallucinations are not real; (3) no additional delusions; and (4) absence of additional hallucinations in the other senses [12].

The patient underwent a neuropsychological examination, including the Mattis Dementia Rating Scale [13] and the version for the blind (MoCA-BLIND) [14] of the Montreal Cognitive Assessment (MoCA) [15]. Considering his visual impairments, all these cognitive test materials were administered verbally, thereby omitting all vision-specific items.

The patient underwent an ophthalmologic examination with, notably, measurements of visual acuity and visual evoked potentials.

#### Control samples

We compared the CBS patient’s resting-state functional and structural MRI data with that of three control groups: 14 late-blind subjects (“LB group”; 7 women; mean age 53 ± 15 years) and two healthy sighted control groups (“C-CBS group” and “C-LB group”) to respectively compare with the CBS patient and the LB subjects. The C-CBS and the C-LB groups respectively included 13 healthy subjects (4 women; mean age 60 ± 10 years) and 13 healthy subjects (7 women; mean age 48 ± 11 years) with normal or normal-corrected vision. Table 1 lists all demographic characteristics and etiologies. All LB subjects developed blindness later in life, after having had a period of a normal visual function, but never experienced any visual hallucinations. All LB subjects had no light perception. The CBS patient and C-CBS group differed significantly for age (t(12) = 2.40; *p* = .03; Sokal and Rohlf [16] t-test modified for single-case studies). The LB group and C-LB group did not differ significantly for age (t(25) = .94; *p* = .35) and gender (*χ*^2^ = .03; *p* = .84). None had a history of psychiatric or neurological episodes. The study was approved by the ethics committee of the Medical School of the University Hospital Centre of Liège (Belgium) for the CBS patients and by the ethics committee of the University of Copenhagen and Frederiksberg (Denmark) for the (C-)LB subjects. Written informed consent was obtained from all the subjects enrolled in the study. The individual in this manuscript has given written informed consent (as outlined in PLOS consent form) to publish these case details. The study was conducted in accordance with the Declaration of Helsinki.

**Table 1:** Demographic details per subject.

Group	Subject	Age	Gender	Etiology of blindness
CBS	1	85	M	Retinitis pigmentosa
LB	1	66	M	Glaucoma
LB	2	64	M	Retinitis pigmentosa
LB	3	51	F	Retinal detachment
LB	4	59	F	Iris infection, cataract
LB	5	44	F	Retinal detachment
LB	6	60	F	Retinal detachment
LB	7	79	M	Retinal detachment
LB	8	57	M	Glaucoma, cataract, retinal detachment
LB	9	40	F	Retinoblastoma
LB	10	74	M	Eye trauma
LB	11	44	M	Retinoschisis
LB	12	25	F	Glaucoma, cataract, retinal detachment
LB	13	43	M	Retinoblastoma
LB	14	39	F	Retinal detachment
C-CBS	1	52	M	-
C-CBS	2	60	M	-
C-CBS	3	62	M	-
C-CBS	4	63	M	-
C-CBS	5	61	M	-
C-CBS	6	70	F	-
C-CBS	7	60	M	-
C-CBS	8	31	M	-
C-CBS	9	65	F	-
C-CBS	10	56	F	-
C-CBS	11	57	F	-
C-CBS	12	72	M	-
C-CBS	13	66	M	-
C-LB	1	52	M	-
C-LB	2	51	F	-
C-LB	3	50	F	-
C-LB	4	53	F	-
C-LB	5	55	F	-
C-LB	6	56	M	-
C-LB	7	59	M	-
C-LB	8	46	F	-
C-LB	9	36	M	-
C-LB	10	61	M	-
C-LB	11	27	M	-
C-LB	12	55	M	-
C-LB	13	28	F	-

M = male; F = female; CBS = Charles Bonnet syndrome; LB = late blind; C-CBS = control-Charles Bonnet syndrome; C-LB = control-late blind

### MRI data

#### Data acquisition

All structural and functional images were acquired on two different magnetic resonance machines of the same manufacturer (3 Tesla Siemens Magnetom TrioTim scanner (Liège) and Siemens AG scanner (Copenhagen)): the CBS patient and controls (C-CBS group) datasets were acquired at the University Hospital Center of Liège, whereas the LB subjects and controls matched for gender, age and handedness (C-LB group) datasets were acquired at the University of Copenhagen. Ultimately, we obtained two independent samples: C-CBS group to compare with the CBS patient and C-LB group to compare with LB group (see Table 1 for more details).

A high-resolution T1-weighted image was acquired for the CBS patient and controls at the University Hospital Centre of Liège (T1-weighted 3D gradient echo images using 120 slices, repetition time = 2300 ms, echo time = 2.47 ms, voxel size = 1×1×1 mm^3^, flip angle = 9 degrees, field of view = 256×256 mm^2^) and for LB subjects and controls at the University of Copenhagen (T1-weighted 3D magnetization-prepared rapid gradient echo sequence “3D MP-RAGE” using 92 slices, repetition time = 1540 ms, echo time = 3.9 ms, voxel size = 0.93×0.93×0.93 mm^3^, field of view = 256 × 256 mm^2^).

Multislices T2-weighted fMRI images were obtained during 10 minutes in CBS patient and C-CBS by using Echo Planar Imaging sequence with axial slice orientation (300 volumes, 32 slices, voxel size = 3.0 × 3.0 × 3.75 mm^3^, repetition time = 2000 ms, echo time = 30 ms, flip angle = 90°, field of view = 384 mm, matrix size (I, J, K) = 64 × 64 × 32, delay = 0, slice order = sequential descending). For LB and C-LB, we used Echo Planar Imaging sequence with axial slice orientation (280 volumes for controls and 270 volumes for LB subjects, 42 slices, voxel size = 3.0 × 3.0 × 3.0 mm^3^, repetition time = 2150 ms, echo time = 2.32 ms, flip angle = 90°, field of view = 192 mm, matrix size (I, J, K) = 64 × 64 × 42, delay = 0, slice order = interleaved ascending). All subjects were instructed to keep their eyes closed and to not think of anything in particular. The CBS patient did not hallucinate during the MRI exam. The first three initial volumes were discarded to avoid saturation effects (as described in [17]).

#### Data pre-processing

Structural (T1-weighted) MRI images in all four datasets were manually reoriented to the anterior commissure/posterior commissure (AC-PC) scheme and segmented (i.e., into grey matter (GM), white matter (WM), cerebrospinal fluid (CSF), skull, and soft tissue outside the brain), subsequently normalized using the segmentation and normalization modules of Statistical Parametric Mapping 8 (SPM8) and VBM/DARTEL, with the standard tissue probability maps and VBM template. The SPM8/VBM8 segmentation was preferred over SPM12 unified segmentation in order to achieve potentially more precise segmentation and normalization using high dimensional DARTEL tissue projection.

For each subject, the volumes of the LGN were estimated bilaterally [18]. The LGN receives afferent input from the retina and projects to primary visual cortex through the optical radiations. In brief, a ROI-based approach combining an automatic segmentation method [19] with probabilistic maps registered to the Montreal Neurological Institute (MNI) space [20–21] was adopted.

For cortical thickness analysis, T1-weighted images were processed using FreeSurfer image analysis suite v5.1.0 (http://surfer.nmr.mgh.harvard.edu/). Technical details have been described previously [22–23]. Automated cortical parcellation and region of interest definition were performed using the Desikan-Killiany Atlas [24], resulting in mean cortical thickness estimations calculated from all vertices within 34 cortical parcellations per hemisphere.

Functional volumes were first manually pre-coregistered with the structural images, and then preprocessed using SPM8. First, the EPI volumes were corrected for the temporal difference in acquisition among different slices using the slice timing correction module with the reference slice set to the first temporal slice, and then the images were realigned to the mean EPI image for head-motion correction.

The mean EPI image across all realigned volumes was then auto-coregistered to the structural image. Then the structural image was segmented into three tissues: GM, WM and CSF in the subject’s space, producing as a by-product of the segmentation the parameters of the transform from the subject’s space to MNI space. This transform was then used to normalize the structural image, the co-registered EPI images and the segmented tissues. Finally, all the coregistered and normalized EPI images were smoothed with an isotropic Gaussian kernel (8 mm full-width-at-half-maximum).

A manual inspection of the whole BOLD timeseries motion was conducted from the SPM motion file to exclude any subject where the translational head displacement was greater than 1 mm, or if the rotational displacement was greater than 0.1 radians. With the aim of reducing loss of signal or whole subjects exclusion due to motion artifacts [25], we used the artifact detection toolbox (NITRC ART, https://www.nitrc.org/projects/artifact_detect/) for artefactual volume detection and rejection using a composite motion measure (largest voxel movement) with a “liberal” threshold (global threshold 9.0, motion threshold 2.0, use scan-to-scan motion and global signal). With this approach, a volume was defined as an outlier (artefact) if the largest voxel movement detected was above the specified thresholds. We subsequently included outliers in the global mean signal intensity and motion as nuisance regressors (i.e., one regressor per outlier in the first-level general linear model). Thus, the temporal structure of the data was not disrupted.

Several parameters were included in a linear regression using CONN v17F and SPM12 to remove possible spurious variances from the data. These were i) six head motion parameters obtained in the realigning step, ii) non-neuronal sources of noise estimated using the anatomical component-based noise correction method (aCompCor; [26]), iii) the representative signals of no interest from subject-specific white matter and cerebral spinal fluid included the top five principal components from white matter and the top five from cerebral spinal fluid mask [27]. Then the residual time series were linearly detrended (no despiking) and temporally band-pass filtered (0.008–0.09 Hz) using CONN’s denoising procedure.

#### Statistical analysis

A T1-based VBM analysis of brain structure (http://dbm.neuro.uni-jena.de/vbm/; executed in MATLAB R2011a Mathworks) was applied to search for potential morphological differences in GM volume, as previously described [17]. The statistical test consisted of a two-sample t-test with assumed independence and equal variance, and with an absolute threshold masking of 0.1 (implicit and explicit masks were disabled) and without overall grand mean scaling. The analyses were performed considering age, gender, and total intracranial volume as covariates. One such test was done separately for each dataset. In the design matrix, a contrast was applied to identify the differences between the CBS patient and C-CBS and then between the LB and C-LB. Because the MRI scans were obtained from two different scanners, the samples were not combined. Results were considered significant at *p*-uncorrected <0.001.

For the average volumes of the LGN, we used a t-test for group comparison.

We used the Single-Bayes procedure [28–29] for statistical analysis of cortical thickness data. In this procedure, a Bayesian inferential method (SingleBayes.exe) was performed to test if the patient’s values are significantly different from the respective values of the LB subjects sample and the controls group. This statistical approach permits to prevent the overestimation of evidence in favor of an effect [30].

We conducted the analysis of the fMRI resting state functional connectivity by using a seed-based correlation analysis (SCA) approach, more specifically seed-to-voxels to cover changes in correlation of the BOLD signal in the whole brain with respect to the specified seed regions. Using SPM12, we used extracts from fMRI BOLD time series from a region of interest (the seed) and measured the temporal correlation between this signal and the time series of all other brain voxels. Two seeds were defined as spheres of 5 mm radius around the peak coordinates of the two main default mode network (DMN) nodes (mPFC: −1, 54, 27 and PCC: 0, −52, 27) taken from [31]. We extracted time series from the voxels in each seed region and then averaged them together to define the DMN region. We investigated the visual networks, including secondary visual cortex and associative visual cortex. For each of them, we defined two seeds as 5mm-radius spheres around peak coordinates of the two main nodes taken from the literature: the secondary visual cortex (–6, -78, -3) (6, -78, -3) and the associative visual cortex (–30, -89, 20) (30, -89, 20) [32]. We also investigated the salience network: the dorsolateral prefrontal cortex (–38, 52, 10) (30, 48, 22) and the thalamus (–12, -18, 6) (12, -18, 6) [33]. Each group was analyzed separately to avoid biases due to different machines and acquisition parameters. We used the averaged time series to estimate whole brain positive and negative correlation r maps, and the t-test contrasts. In the design matrix, we applied a contrast to identify the differences between the CBS patient and C-CBS and then between the LB and C-LB groups. Age and gender were further added as regressors.

Statistical results were considered significant with multiple comparison correction at the topological level with non-parametric permutation test cluster-mass *p*-FWE < 0.05 and primary voxel-wise threshold *p*-uncorrected < 0.001 (see [34–35]) using CONN 17f with the generalized permutation of residuals patch.

## Results

### Neuropsychological and ophthalmological examinations

The Mattis Dementia Rating Scale and the MoCA-BLIND scores were respectively 79/79 and 22/22. These results allow us to consider the patient’s cognitive and global functioning as normal.

The patient was diagnosed with an ophthalmologic disease and revealed retinal atrophy in both eyes. He had also a decompensated corneal grafting in the left eye. He had undergone a cataract operation of both eyes. Flash visual evoked potentials were flat. At the time of the clinical assessment the patient could perceive light in both eyes.

### MRI results

#### Structural MRI

**Fig 1:**
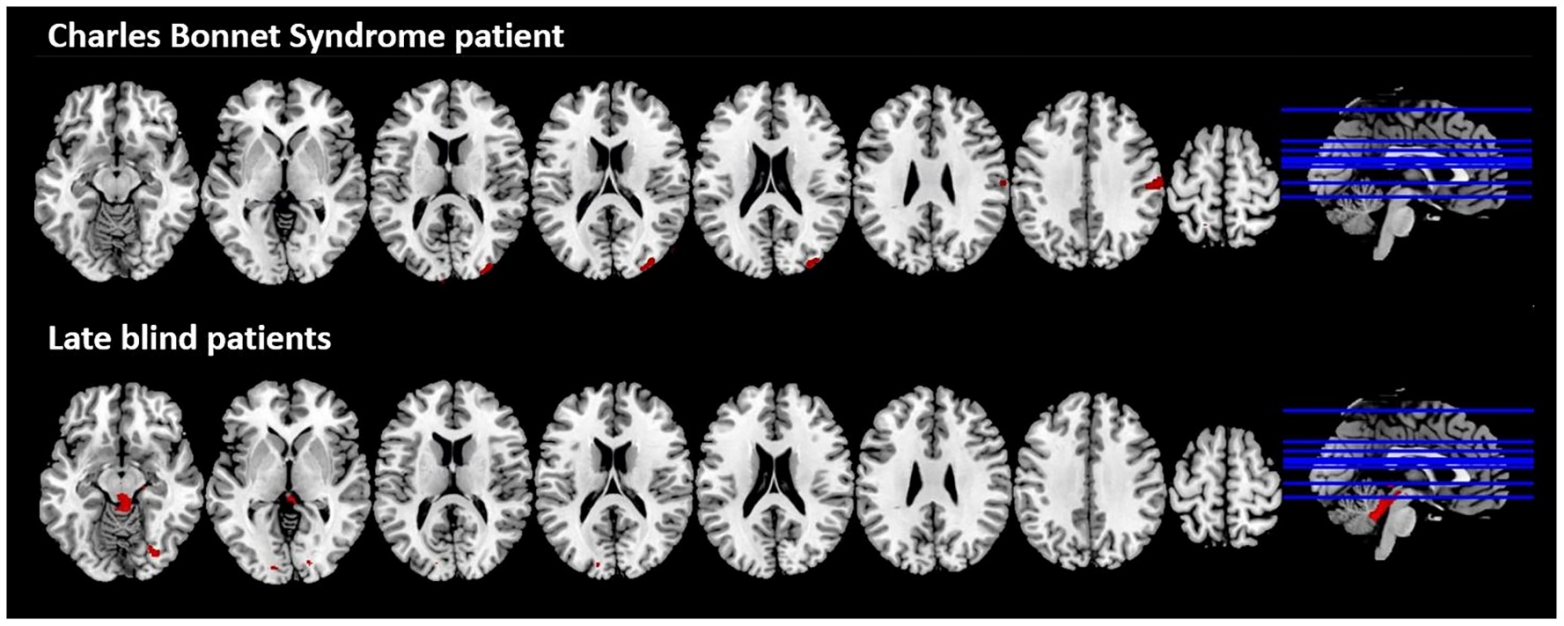
VBM-based grey matter decreases in the CBS patient compared to C-CBS group (top) and in the LB group compared to C-LB group (bottom); multislice view of the regions overlaid on the MNI template; *p*-uncorrected <0.001.

The CBS patient showed decreased GM volume in the occipital lobe (more precisely in the middle occipital gyrus and in the cuneus), in the postcentral gyrus and in the superior parietal lobule, as compared with C-CBS (Fig 1, upper row). In the LB group, we also identified reduced GM areas in the occipital lobe (more precisely in the lingual gyrus and in the middle occipital gyrus) and in the left and right cerebellum, as compared with healthy controls (Fig 1, lower row).

The average volumes of the left and right LGN were respectively 72.2 ± 36.3 mm^3^ and 94.34 ± 37.5 mm^3^ in sighted controls, compared to 54.2 mm^3^ and 63.28 mm^3^ in CBS patient (*p* = .18 for left LGN and *p* = .06 for right LGN). In LB subjects, the average volumes for the left and right LGN were respectively 53.47 ± 26.5 mm^3^ and 58.2 ± 22.6 mm^3^. A group comparison suggested that the LGN volumes did not differ between the CBS patient and LB subjects (for left LGN: *p* = .48 and for right LGN: *p* = .38).

Additionally, we evaluated separately the cortical thickness in the CBS patient versus the C-CBS controls and versus those of LB subjects. The CBS patient showed significantly lower cortical thickness than controls in several areas, including visual (e.g., bilateral pericalcarine, cuneus, fusiform gyrus, and lateral occipital), and associative areas (e.g., pars opercularis, superior parietal, superior temporal and insula parcels; *p* < 0.0008, post-hoc Bonferroni correction; see Table 2 for a complete list).

**Table 2:** Cortical thickness differences between CBS patient and controls.

	Group		
Brain regions	CBS patient	C-CBS group	*p* value
L cuneus	1.27	1.87	.0008
R cuneus	1.27	1.92	.0006
L fusiform	1.70	2.77	.0003
R fusiform	1.59	2.74	.0002
L inferior parietal	1.47	2.50	.00002
R inferior temporal	1.64	2.87	.0001
L lateral occipital	1.48	2.30	.0006
R lateral occipital	1.54	2.32	.0009
L lateral orbitofrontal	1.67	2.64	.0005
L lingual	1.51	2.04	.00005
L middle temporal	1.65	2.90	.00004
L paracentral	1.50	2.38	.0005
L pars opercularis	1.64	2.58	.0005
R pars opercularis	1.49	2.60	.0002
L pars triangularis	1.48	2.46	.00003
L postcentral	1.29	2.13	.0006
L precentral	1.49	2.58	.0002
L precuneus	1.53	2.43	.00003
L rostral middle frontal	1.58	2.41	.0005
R superior frontal	1.53	2.70	.0002
L superior parietal	1.29	2.24	.000007
R superior parietal	1.24	2.22	.00008
L superior temporal	1.61	2.86	.00001
R superior temporal	1.69	2.85	.0005
L supra marginal	1.51	2.62	.0001
L insula	1.90	3.07	.00001
R insula	2.29	3.11	.0006

L = left; R = right; CBS = Charles Bonnet syndrome

Significant differences in regional cortical thickness between the CBS patient and LB subjects are shown in Table 3. Noteworthy, the CBS patient showed significantly lower cortical thickness than LB subjects in several brain regions, mainly devoted to the processing of multimodal associative information (e.g., bilateral fusiform, inferior parietal, inferior temporal, lateral orbitofrontal, middle temporal, paracentral, pars opercularis, precuneus, superior frontal, superior parietal, and superior temporal parcels; *p* < 0.0008, post-hoc Bonferroni correction).

**Table 3:** Cortical thickness differences between CBS patient and LB subjects.

	Group		
Brain regions	CBS patient	LB subjects	*p* value
R caudal middle frontal	1.55	2.52	.0001
R cuneus	1.27	1.85	.0004
L fusiform	1.70	2.66	.0004
R fusiform	1.59	2.69	.000004
L inferior parietal	1.47	2.47	.0000008
R inferior parietal	1.53	2.51	.00001
L inferior temporal	1.65	2.84	.0000002
R inferior temporal	1.64	2.89	.000001
L lateral orbito frontal	1.67	2.68	.0001
R lateral orbito frontal	1.89	2.64	.0003
L middle temporal	1.65	2.91	.00006
R middle temporal	1.83	2.95	.00002
L para central	1.50	2.40	.0005
R para central	1.41	2.41	.00007
L pars opercularis	1.64	2.51	.0002
R pars opercularis	1.49	2.46	.00002
L pars triangularis	1.48	2.39	.0004
R posterior cingulate	1.77	2.45	.0007
L pre central	1.49	2.55	.00002
R pre central	1.57	2.48	.0001
L pre cuneus	1.53	2.41	.0001
R pre cuneus	1.54	2.40	.00008
L rostral middle frontal	1.58	2.42	.0004
R rostral middle frontal	1.57	2.37	.00005
L superior frontal	1.67	2.75	.00009
R superior frontal	1.53	2.70	.000008
L superior parietal	1.29	2.19	.00001
R superior parietal	1.24	2.20	.00003
L superior temporal	1.61	2.77	.000008
R superior temporal	1.69	2.78	.000008
L supra marginal	1.51	2.54	.00001
R supra marginal	1.58	2.56	.00009
L insula	1.90	2.95	.00001

L = left; R = right; CBS = Charles Bonnet syndrome; LB = late-blind

#### Resting-state fMRI

No significant differences were detected between the CBS patient and C-CBS subjects in DMN connectivity. In contrast, LB subjects showed increased resting-state negative connectivity between the DMN and a cluster including the right occipital fusiform gyrus compared to C-LB group. S1 Fig shows the DMN connectivity of both the C-LB and C-CBS groups.

**Fig 2:**
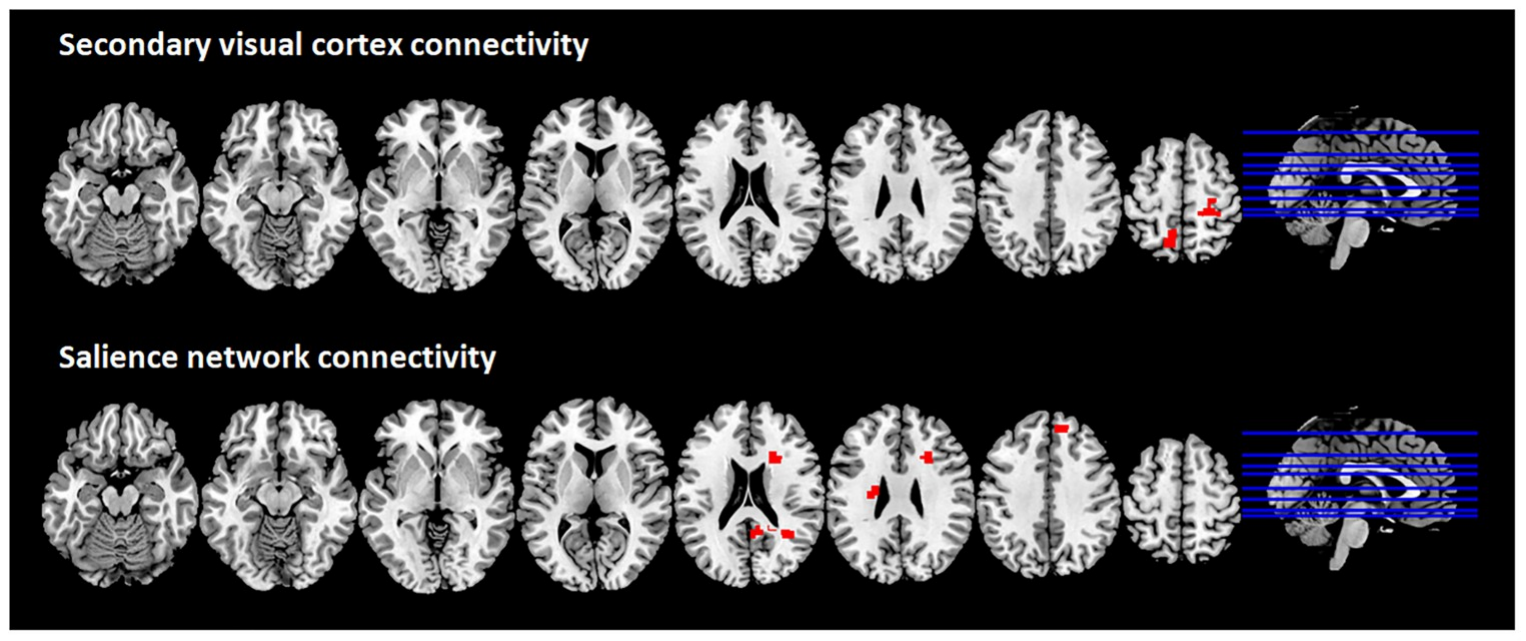
Brain regions showing increased functional connectivity with the secondary visual cortex and the salience network in the CBS patient compared to C-CBS group; multislice view of the regions overlaid on the MNI template; topological non-parametric correction at cluster-mass *p*-FWE <0.05 and primary threshold *p*-uncorrected <0.001.

The CBS patient showed increased connectivity as compared to controls between the secondary visual cortex and two clusters in the left precuneus and the right postcentral gyrus (Fig 2, upper row). No differences were detected between LB and their respective controls regarding the secondary visual cortex connectivity.

The CBS patient displayed a significant increase of positive connectivity between the salience network and three clusters compared to the C-CBS group (Fig 2, lower row): the middle temporal gyrus right, the medial frontal gyrus right and the caudate nucleus left. LB subjects showed significant increased positive connectivity between the salience network and a cluster in the right inferior parietal lobule and the right insula. No differences were detected.

## Discussion

In the present study, a patient without any history of psychiatric disorders developed visual hallucinations after becoming totally blind was investigated by structural and resting state functional MRI. The patient showed the essential features and conditions of CBS. The aim of this study was to examine purported structural and functional brain changes associated with visual hallucinations and the loss of vision.

In order to disentangle brain modifications due to the late visual loss from those underpinning complex visual hallucinations typical of CBS, we included a group of LB subjects without any visual hallucinations. In line with our hypothesis, our CBS case showed regional reductions in cortical thickness when compared to control subjects, encompassing primary visual, associative and multimodal cortices. However, many of these differences disappeared in the comparison of CBS with the LB group, suggesting that these structural alterations are primarily related to blindness. Indeed, when comparing the CBS patient with the LB subjects, only the reductions in cortical thickness in associative and multimodal cortices were significant, suggesting a role for these areas in the genesis of visual hallucinations. Moreover, consistent with previous structural imaging studies reporting atrophy in the occipital lobes in CBS patients (e.g., [3,8]), we observed VBM-based GM reduction (after controlling for age, gender, and total intracranial volume) in the middle occipital gyrus and in the cuneus in the studied CBS patient, compared to a control group. This partly differed from the GM reductions found in LB subjects that covered the lingual gyrus and the middle occipital gyrus, as well as the left and right cerebellum, compared to a control group.

In order to investigate if these structural alterations were paralleled by functional alterations, we carried out a resting state functional connectivity study. Our analysis showed a functional reorganization of the brain connectivity between regions involved in self-awareness and in visual and salience processing, when observing the respective findings obtained with the CBS patient and the LB subjects. The precuneus showed increased connectivity with the secondary visual cortex in the CBS patient. This is consistent with Kazui and colleagues’ [3] study demonstrating hyperactivity in the primary and secondary visual cortices in CBS, assuming this to be a consequence of deafferentiation [36]. Indeed, these authors suggested a fundamental dysfunction of the secondary visual cortex in CBS, accompanied by transient cortical activation of the inferior lateral temporal cortex (principally the fusiform gyrus region) during the emergence of visual hallucinations [3]. Nonetheless, the causes of the activity and connectivity changes in CBS patients are still unknown.

In this study, we observed an increase of resting-state negative connectivity between the DMN and a cluster including the occipital fusiform gyrus in the LB subjects, whereas no such significant functional connectivity changes were identified between the CBS patient and the associated controls. This might suggest that a functional connectivity reorganization in the visual network is taking place for LB subjects but not in CBS. The fusiform gyrus is known to support hallucinations of faces in CBS patients [37] and other studies demonstrated increased activity in this ventral occipital cortex during visual hallucinations in CBS patients [9]. However, further studies should investigate functional connectivity during resting state (i.e., without active hallucinations) in a greater number of CBS patients, in particular in the visual network, to assess the resting state connectivity changes that characterize this population. More generally, the literature reveals that resting state (without hallucinating) can show abnormal functioning in subjects who report hallucinatory experiences (see [38] for a review).

The present results offer evidence of cortical network reorganization between the salience network and some specific (but not identical) regions in both the CBS patient and the LB as compared with respective matched sighted controls. Some studies have associated both activity and connectivity changes in the salience network, and more specifically in the dorsal prefrontal cortex, with the presence of phantom percepts (e.g., [39–41]) and the propensity to hallucinate [39,42]. Indeed, this network appears to play a key role in the detection of the salience of external and internal stimuli [43]. In our CBS patient, we observed a functional reorganization of the brain connectivity between regions involved in salience processing and some specific regions subserving visual perception and multimodal sensory integration, such as the middle temporal gyrus [44].

It has been debated to which extent CBS could be caused by sensory deprivation as a result of eye deficits or some brain lesions [45]. Some authors have suggested that the loss of vision may lead to deafferentation of the visual association cortex [46], leading to disinhibition and spontaneous development of hallucinations because of visual cortex activation [9]. Another theory, the ‘release phenomenon’ theory, claims that removal of visual input results in reduced cortical inhibition and thus releases percepts stored in the brain, resulting in visual hallucinations [47]. In individuals with CBS, visual loss due to ophthalmic pathology leads to a state of sensory deprivation that releases the visual cortex from regulation by externally produced visual stimuli, which may result in hallucinations (i.e., an inappropriate pattern of cortical excitation). The pathophysiology of CBS shares similarities with the phantom limb pain phenomenon [48]. Consequently, not only vision deficit, but also dysfunction in the primary and secondary visual systems could be major contributors to the occurrence of visual hallucinations in CBS patients. The reasons why some patients with visual deficits develop hallucinations and CBS, and other do not, is yet unknown. No theory can explain why CBS is so rare in the LB population suffering from visual deprivation of the occipital cortex. Also, whether the connectivity changes observed in our CBS patient are the *primum movens* to the hallucination or only a result of them is not clear now.

The present study is not free of limitations. The main limits are represented by the small number of patients (i.e., 1 CBS patient and 14 LB subjects) and the fact that CBS and LB groups were examined using two different scanners. In addition, the LB group include different eye pathologies. For these reasons the current findings cannot be generalized to the entire LB population and should be interpreted cautiously. The age difference between the CBS patient and the comparison groups is also a limitation, however, we used the factor age as a regressor in the present analyses to limit the potential bias. Further studies should investigate functional connectivity in a greater number of CBS patients and with LB subjects acquired on the same scanner; nonetheless, this syndrome is relatively rare. Although hallucinations in CBS are visual in the majority of cases, few CBS patients also report somatosensory (or other modalities) hallucinations [49–50], which are worth to be investigated. The presence of hallucinations in other modalities occurring in CBS patients however remains controversial in the literature [49] and it is not clear to what extent eye disease may play a part in their emergence. More generally, further studies are needed to determine a more detailed neurobiological explanation of CBS.

In conclusion, our overall resting state investigation showed brain connectivity changes specific to the CBS case, notably in visual related and salience networks. A functional connectivity reorganization following alterations in visual brain structures may contribute to visual hallucinations in CBS. Nonetheless, the explanation of this phenomenon remains incomplete. CBS symptoms can last for many years and have negatives consequences in around a third of CBS population [51], thus a better understanding of this pathology might enhance the healthcare provided to this population and their quality of life. From a clinical perspective, a better understanding of this syndrome is essential since, for example, a recent study suggests that patients with CBS may present an increased risk of subsequently developing dementia, when combined with Mild Cognitive Impairment and partial impairment of insight [52]. Indeed, so far the interpretation of the connectivity changes, as well as their clinical significance, is still not understood. The present findings have important implications not only for the more general understanding of the functioning of the human brain, but also for our understanding of how hallucinations and blindness can change brain function and structure.

## Supporting information

S1 Fig Default mode network (DMN) connectivity in the C-LB group and in the C-CBS group. Cluster-mass *p*-FWE <0.05 and primary threshold *p*-uncorrected <0.001.
(TIFF)
