# Supplementary figures and images for: Resting-state functional connectivity and cortical thickness characterization of a patient with Charles Bonnet syndrome
Source: PLoS One. 2019 Jul 18;14(7):e0219656. doi: 10.1371/journal.pone.0219656 (PMC6638931; doi:10.1371/journal.pone.0219656)

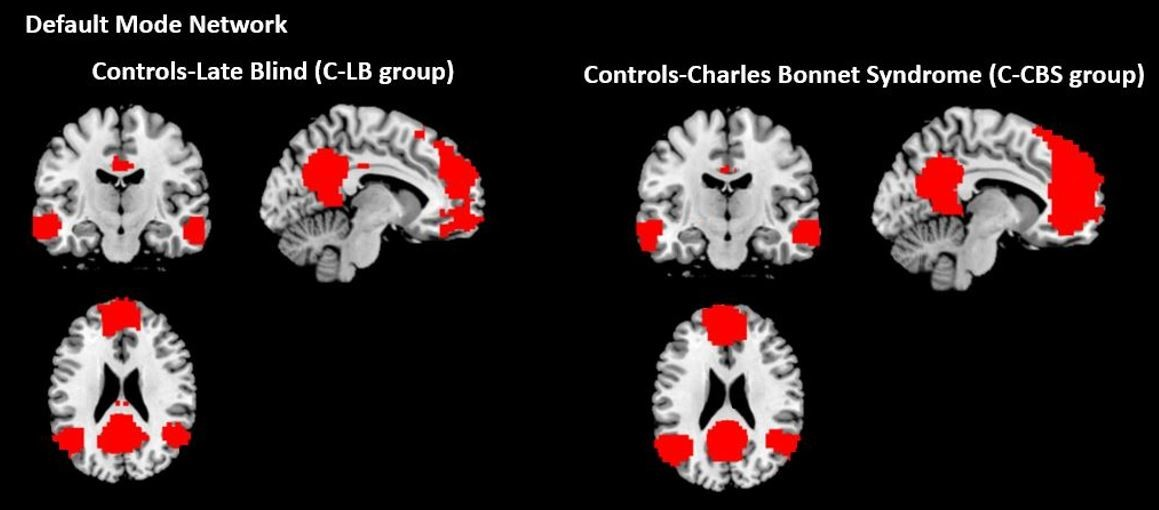

Supplement: S1 Fig — Cluster-mass p-FWE <0.05 and primary threshold p-uncorrected <0.001. (TIFF) [file pone.0219656.s001.tiff]
